# The wheat clock strikes a balance across subgenomes to regulate gene expression

**DOI:** 10.1371/journal.pbio.3001825

**Published:** 2022-10-14

**Authors:** Kathleen Greenham

**Affiliations:** Department of Plant and Microbial Biology, University of Minnesota, Saint Paul, Minnesota, United States of America

## Abstract

Increasing crop yields is complicated by the polyploid nature of our major crops. This Primer explores the implications of a PLOS Biology study that provides a transcriptomic view of the influence of the circadian clock on agriculturally relevant traits in the polyploid bread wheat.

## Teaser

Increasing crop yields is complicated by the polyploid nature of our major crops. A recent *PLOS Biology* study provides a transcriptomic view of the influence of the circadian clock on regulating agriculturally relevant traits in the polyploid bread wheat.

Wild ancestors of modern crops are barely recognizable due to the incredible changes made through domestication. Humans transitioned from hunter-gatherers to farmers 10,000 years ago, and 6,000 years later major crops like rice, wheat, and maize had been domesticated [1]. Today, crop yield is threatened by climate change, and the Food and Agriculture Organization estimates suggest we need a 70% increase in yield by 2050 to meet growing demand. The circadian clock was an important target of crop domestication due to its regulation of yield-related traits such as flowering time, growth, stress responses, and metabolism [2–4]. Circadian oscillators are found in all kingdoms of life providing an internal timekeeper that coordinates and responds to environmental signals [5]. Our understanding of the plant clock is primarily from work in the model dicot plant *Arabidopsis thaliana*. With many major crops being monocots, a recent study [6] set out to characterize the circadian transcriptome of the bread wheat *Triticum aestivum*, one of the most important and widely grown crops globally. Roughly one-third of expressed genes were classified as clock regulated in *T*. *aestivum* with period lengths longer and with a higher variance than *Arabidopsis*, *Brassica rapa* (field mustard), *Glycine max* (soybean), and *Brachypodium distachyon* (temperate grass model), suggesting that circadian clock features in wheat are distinct.

**Fig 1:**
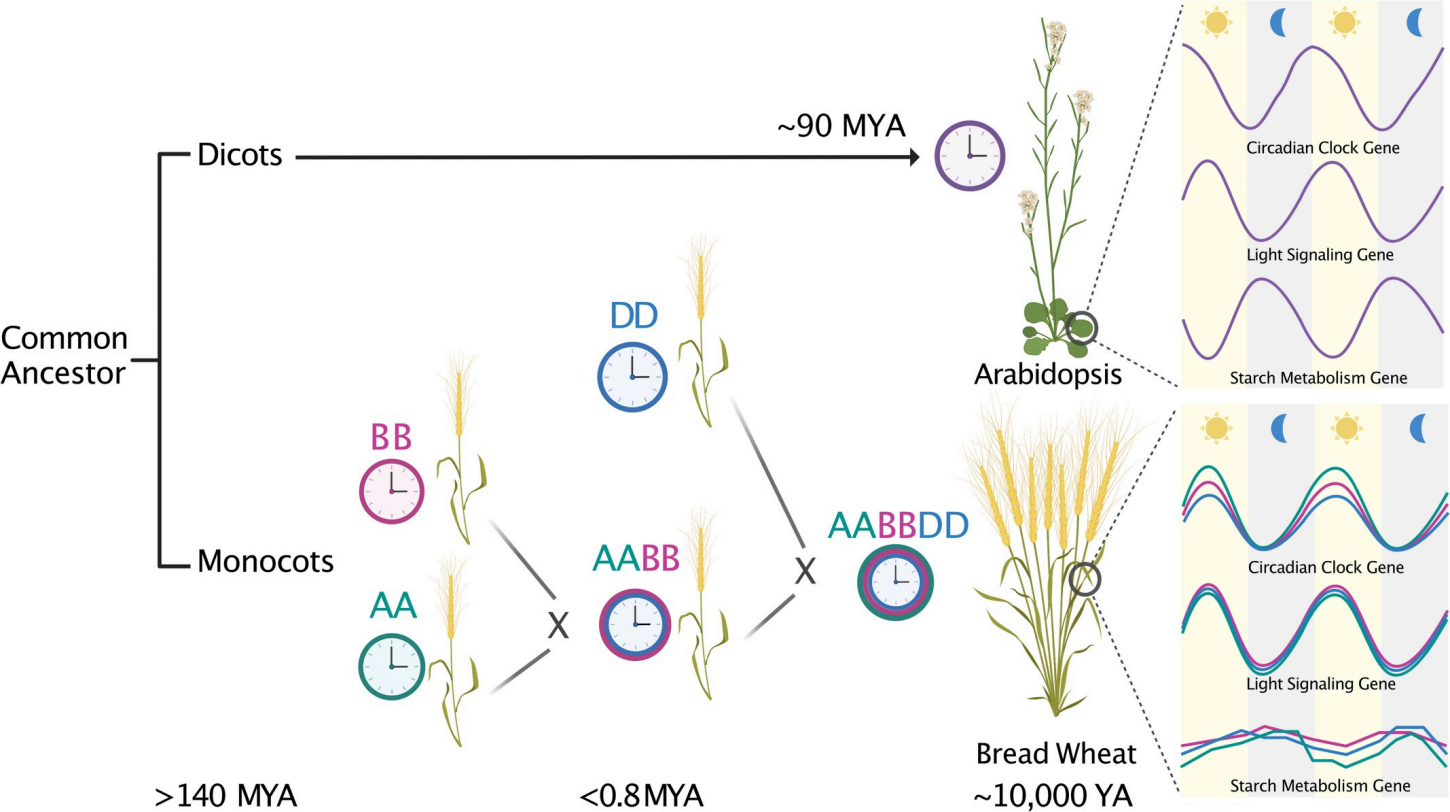
Clock-controlled pathways in *Arabidopsis* and the hexaploid wheat are largely conserved. Following the divergence of monocots and dicots over 140 million years ago (MYA), the ancestors of bread wheat evolved. Interspecific hybridization between the tetraploid (AABB) and diploid (DD) ancestors gave rise to the hexaploid (AABBDD) bread wheat we eat today. Comparisons of clock regulation of gene expression with *Arabidopsis* highlight examples of conservation and divergence in clock control of critical biological pathways. The temporal patterns of expression for circadian clock and light signaling genes are largely conserved, although clock genes retained in 3 copies often varied in amplitude across the subgenomes and had a 3.5-h longer mean period than *Arabidopsis*. Starch metabolism has diversified in terms of clock control of transcript abundance for genes within the pathway suggesting altered regulation of this process. Created with BioRender.com.

Essential genes for clock function (the core) generate interlocking transcriptional/translational feedback loops that lead to time-of-day–specific expression of genes, proteins, enzymes, and metabolites [5]. Core clock components act primarily as repressors, inhibiting each other to maintain time-of-day–specific expression. Divergence between monocots and dicots occurred over 140 million years ago, yet much of the core is conserved [2]. Alleles of many of these genes are associated with key agronomic traits including photoperiodic flowering, heading date, plant height, and pathogen defense [2,3]. One of the challenges in translating knowledge from *Arabidopsis* to crops is their polyploid nature from recurrent whole-genome duplications [7]. As a result, many crops have multiple orthologs of the same *Arabidopsis* gene. Bread wheat (*T*. *aestivum*) is a hexaploid (AABBDD) that formed around 10,000 years ago from the interspecific hybridization between a tetraploid (AABB) and diploid (DD) ancestor [8]. The analysis by Rees and colleagues reveals that hexaploid wheat integrates components of each subgenome (A, B, and D) into a circadian clock with control over critical physiological processes like the distantly related *Arabidopsis* clock (Fig 1).

With 51.7% of genes present in 3 copies (triads) in *T*. *aestivum*, Rees and colleagues tested whether there was evidence of 1 subgenome clock being more transcriptionally active or having unique circadian properties. A previous study in wheat found that 72% of triads had equivalent transcript abundance levels or were “balanced” [9]. Rees and colleagues performed a circadian time course RNA-sequencing experiment under constant light and temperature where rhythms in gene expression are driven solely by the clock. Rhythmicity analysis identified significantly rhythmic genes and assigned period (time between peaks), phase (time of peak), and amplitude values. Circadian imbalance was classified based on differences in these parameters for each triad. For the 16,359 expressed triads, more than 3 times as many were imbalanced as balanced, with rhythmicity accounting for the most variation. Interestingly, when assessing imbalance on the expression level as done previously [9], the level of imbalance changed depending on the time point emphasizing the importance of studying expression dynamics over time. It should be noted that these 2 studies were done in 2 different cultivars of wheat. Unique to the balanced triads was overrepresentation of gene functions related to photosynthesis and metabolic energy generation, which were also found among *B*. *rapa* paralogs with similar circadian expression pattern [10].

Consistent with studies in *B*. *rapa* [10], the balanced rhythmic triads were expressed at uniformly higher levels than even the highest expressed imbalanced triads, suggesting that all subgenome copies are contributing to clock function. A thorough comparison of circadian clock gene expression between wheat and *Arabidopsis* revealed most genes maintained a similar phase although they varied in period length. Gene coexpression networks were constructed to identify modules, or clusters of genes with similar expression patterns. Correlated modules with similar phase shared more Gene Ontology (GO) terms in common than modules that were out of phase.

Among all rhythmic genes between *Arabidopsis* and wheat, photosynthesis, response to abiotic stress, and macromolecule biosynthesis were enriched, supporting the evolutionary conservation of clock-controlled functions. These traits are especially relevant for current wheat breeding efforts that prioritize increasing photosynthetic capacity, yield, and stress tolerance [3,8]. For each of these pathways, examples could be found where specific genes had conserved phasing of expression in wheat and *Arabidopsis*, and examples of unique phasing of certain wheat homeologs, highlighting differences in circadian control (Fig 1). A pathway exhibiting more diverged clock regulation was starch metabolism, a process that is critical for plant growth [11]. In *Arabidopsis* and wheat, starch accumulates during the day and is degraded at night at a rate that is dependent on the length of the night [11,12]. In *Arabidopsis*, this process is clock controlled [11], and genes involved in starch biosynthesis and catabolism are circadian regulated. In wheat, there was much more variation in rhythmicity and phasing for genes in both the biosynthesis and catabolic steps including loss of rhythmicity for many homeologs suggesting that there is less constraint at the transcript level. Whether the proteins are rhythmic or this indicates less circadian control of this pathway is unknown.

Rees and colleagues provide new insight into the complexities of circadian regulation in an allopolyploid crop. This represents just 1 cultivar within *T*. *aestivum*, a crop that is grown between 67° N and 45° S with more than 560,000 cultivars adapted to different environmental regions that include winter and spring types [13]. How have their circadian networks been shaped to accommodate such wide geographic ranges? Based on studies in tomato, soybean, and barley, we can expect to find variation in circadian properties across latitudinal clines [3]. If this holds true in wheat, are the circadian imbalanced and balanced triads conserved across the species or do we see intraspecific variation in the subgenome contributions to the circadian network? Additional time course datasets across diverse cultivars will be critical to identifying targets specific to the geographical location and in developing models to predict what effect integrating new traits will have on the underlying circadian network. If the 140 million-year conservation of clock control over these critical biological processes has taught us anything, it is that we can no longer ignore the clock.
